# Semi-Monthly Meeting, Rochester Medical Society

**Published:** 1874-09

**Authors:** J. O. Roe

**Affiliations:** Secretary


ART. IV.—*Semi-Monthly Meeting, Rochester Medical Society.*
Reported by J. O. ROE, M. D., Secretary.

Dr. W. S. ELY, President, in the chair.

After the preliminary business, the subject for the evening was then offered by Dr. E V. STODDARD.

Subject: "The most recent Views of the Physiological and Therapeutical Properties of Cinchona and its Alkaloids."

He stated that we recognize cinchona bark and quinia as possessing properties antiseptic, antiperiodic and antiphlogistic.

(1.) Antiseptic. From its peculiar influence upon the microzymes upon which the putrifactive process is so largely dependent, including the various forms of bacteria, bacillus, &c.

The action of quinia upon the microzymes is inhibitory, not toxic. Hence it may check septic changes, but not destroy the organisms upon which they depend.

Upon the penicilium quinia exerts no apparent influence, and hence has no appreciable modifying power upon fermentation.

(2.) Antiperiodic. The attempt to explain the cause of periodic disorders by this agency of zymotic organisms can not be established, since such organisms have not yet been detected in the blood. Hence the surmise that quinia proves curative in these diseases by destroying such organisms is merely speculative.

Yet it is desirable to establish the fact of the similarity, if there be any, in the antiseptic and antiperiodic properties of this drug.

He remarked upon the discovery of animal quinoidine by Bence, Jones and Dupri—experiments showing an animal fluorescent substance to be identical chemically in constitution with quinia, and the existence of this substance as a normal component element of the blood. Dr. Stoddard also pointed out the significance of its presence or absence in disease as explaining the curative action of quinia in some forms of anæmic and periodic diseases.

(3.) Antiphlogistic. Prof. Bintz was the first to point out the toxic action of quinia upon the white corpuscles; hence the significance of its effect in checking the movements of the wandering cells in inflammatory diseases.

This migration of leucocytes in inflammation, and the power of quinia to arrest them, is clearly proven by Conheim's experiments.

In the discussion that followed, Dr. Moore referred to the importance of the fluorescent test, and inquired whether any other substance or substances could be confounded with that of quinia or animal quinoidine.

Dr. Stoddard replied, that by spectral analysis they had been found identical, and no known substance produced the same spectral lines.

Considerable discussion then followed upon the subject of fluorescence.

After a lengthy discussion of the therapeutic properties of quinine, the Society adjourned to meet at the next regular meeting in two weeks.

